# Alcohol Exposure Induces Depressive and Anxiety-like Behaviors via Activating Ferroptosis in Mice

**DOI:** 10.3390/ijms232213828

**Published:** 2022-11-10

**Authors:** Congyue Xu, Qi Xiong, Xiang Tian, Wei Liu, Binlian Sun, Qin Ru, Xiji Shu

**Affiliations:** 1 Wuhan Institutes of Biomedical Sciences, School of Medicine, Jianghan University, Wuhan 430000, China; 2 Department of Health and Physical Education, Jianghan University, Wuhan 430000, China

**Keywords:** alcohol, ferroptosis, synaptic plasticity, depression, anxiety

## Abstract

Alcohol use disorder (AUD) is a global public health problem and is frequently comorbid with mental disorders, including anxiety and depression. Ferroptosis is an iron-dependent cell death, which is involved in the pathological process of various diseases such as neurodegenerative diseases, but the role of ferroptosis in the mediation of AUD and its induced mental disorders is unclear. In this study, we aimed to investigate whether ferroptosis was involved in alcohol-induced depressive and anxiety-like behaviors in mice. Following an 8-week period of intermittent alcohol exposure, the alcohol group showed noticeable depressive and anxiety-like behaviors. In addition, nissl staining revealed that alcohol exposure induced neuron damage in the hippocampus (Hip) and prefrontal cortex (PFC) of mice. The levels of synapse-related proteins were significantly reduced in the alcohol group. Iron staining demonstrated that alcohol increased the number of iron-positive staining cells. The protein expression of the transferrin receptor (TFRC) was increased, and the expression of glutathione peroxidase 4 (GPX4) was decreased, respectively, in the alcohol group. Furthermore, the ferroptosis inhibitor ferrostatin-1 significantly prevented alcohol-induced neuron damage and enhanced the expression of *N*-methyl-d-aspartic acid (NMDA) receptor 2B (NR2B), α-amino-3-hydroxy-5-methyl-4-isoxazole-propionic acid (AMPA) receptor 1 (GluA1) and GPX4 in vitro. These results indicated that alcohol exposure could induce depressive and anxiety-like behaviors, and that this effect may occur via activating ferroptosis.

## 1. Introduction

Alcohol use disorder (AUD) is a kind of chronic recurrent disorder which is one of the most common substance use disorders. Alcohol abuse and dependence are two types of AUD. AUD is a devastating condition affecting over 5.1% of the global burden of disease, and causing 300 million deaths worldwide [1]. Drinking tolerance is reduced in people with AUD, leading to physiological dependence, with symptoms such as significant irritability, sleep disorders, autonomic nervous dysfunction, anxiety and depression. Despite the negative implications to their personal and professional lives, they continue to drink. According to epidemiological research, up to 40% of AUD patients have a concomitant mood disorder, while up to 35% of anxiety disorder patients have AUD [2]. According to a prior study, those who are addicted to alcohol are twice as likely to develop depression than people who are not addicted to alcohol [3]. AUD interacts with depression and anxiety, and the etiology of the comorbidity is complex; antidepressant drugs have been the focus of most drug trials for AUD co-occurring with depression.

Synapses are the basic structure of information transmission between neurons [4]. Synaptic plasticity refers to the adjustable strength of the connections between synapses, that is, the characteristic or phenomenon of lasting changes in the morphology and function of the synapse [5]. Glutamic acid ionic receptors, *N*-methyl-d-aspartic acid (NMDA) receptors, and α-amino-3-hydroxy-5-methyl-4-isoxazole-propionic acid (AMPA) receptors play important roles in maintaining synaptic plasticity. The *N*-methyl-d-aspartic acid receptor (NMDAR) is the key to mediate synaptic plasticity [6]. α-amino-3-hydroxy-5-methyl-4-isoxazole-propionic acid receptor (AMPAR) is the central receptor responsible for synaptic transmission and plays an essential role in the synaptic plasticity [7,8,9] and cell death caused by neurological diseases and functional disorders [10,11]. AMPAR can be involved in the pathogenesis of depression by altering synaptic plasticity [12,13]. Gene expression, density, and function of NMDAR and AMPAR were abnormal in patients with depression [14,15]. In recent years, the mechanism of synaptic plasticity has become the focus of antidepressant drug research. In 2019, United States Food and Drug Administration (FDA) approved Spravato, an NMDA receptor antagonist of glutamate, for the treatment of treat-resistant depression, which was a breakthrough in antidepressant research [16]. Wills et al. found that alcohol affected the expression of synapse-related proteins in the hippocampus (Hip) of long-term depressed (LTD) mice [17]. Similar to clinical findings, the expression of synapse-related subunits and proteins, including AMPAR subunits (GluA 1/2 and 3), NMDAR subunits (GluN1 and N2B), other synaptic proteins, (for example, synapsin1 (SYN1) and post-synapticdensity95 (PSD95)) were reduced in depressed rodents induced by chronic stress [18,19,20]. Therefore, the changes in synaptic plasticity may occur in instances of anxiety and depressive-like behaviors induced by alcohol abuse.

Iron is an essential element for all life forms on earth which is involved in oxygen transport, tissue respiration, hematopoietic function, and other important biochemical processes based on its highly efficient electron transfer properties [21]. However, iron overload also leads to ferroptosis, characterized by phospholipid peroxidation [22]. Alcohol affects body iron metabolism and accelerates iron overload [23]; even modest alcohol intake improves liver iron levels [24]. However, it is unknown whether ferroptosis exists in the Hip and prefrontal cortex (PFC), and the role of ferroptosis in alcohol-induced anxiety and depression is not clear.

We hypothesized that ferroptosis could be involved in alcohol-induced depression and anxiety based on these findings. In this study, we used an intermittent access to alcohol (IAA) model to see if alcohol exposure causes negative emotional behavior. We also analyzed the expression of synapse-related proteins and iron metabolism-related proteins in vivo and in vitro, hoping to find a new pathophysiology for alcohol-induced depression and anxiety.

## 2. Results

### 2.1. Alcohol Exposure Induced Depressive and Anxiety-Like Behaviors in Mice

**Figure 1:**
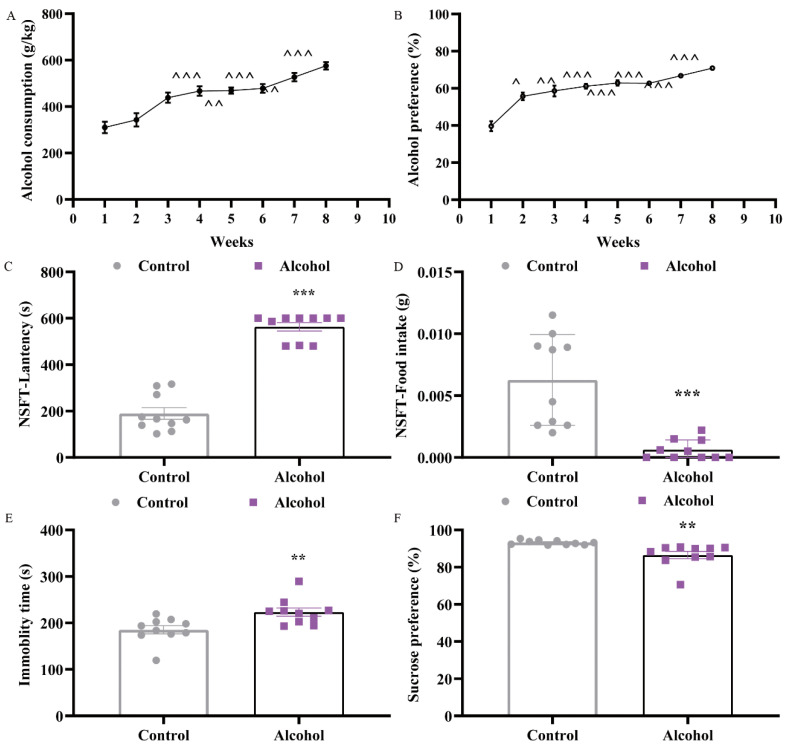
Alcohol exposure induced depressive-like behaviors in mice. (**A**) Alcohol consumption in mice across 8 weeks of alcohol, (**B**) alcohol preference in mice across 8 weeks of alcohol, (**C**) immobility time of mice in tail suspension test, (**D**) latency to feed in novelty-suppressed feeding test, (**E**) the food intake in novelty-suppressed feeding test, and (**F**) the sucrose preference of mice in sucrose preference test. Data are presented as the mean ± SEM, and one-way repeated measures ANOVA was performed for alcohol consumption and preference data with Sidak’s post hoc test. *n* = 10 for per group, ^ *p* < 0.05, ^^ *p* < 0.01, ^^^ *p* < 0.001 compared with week 1. Student’s *t* test was applied to other animal experiments, ** *p* < 0.01, *** *p* < 0.001 vs. control group.

Across the 8 weeks of IAA to build the alcohol dependence mouse model, alcohol consumption and alcohol preference increased significantly with increasing drinking days. Results showed that alcohol consumption in weeks 2~8 were markedly higher than that in week 1, and weeks 4~8 saw significantly increased levels (t_4_ = 8.533, *P*_4_ = 0.0004; t_5_ = 7.422, *P*_5_ = 0.0011; t_6_ = 8.749, *P*_6_ = 0.0003; t_7_ = 7.232, *P*_7_ = 0.0014; t_8_ = 9.76, *P*_8_ = 0.0001) (Figure 1A). Moreover, the alcohol preference of mice in the alcohol group was 39.66% in the first week and increased to 70.89% at the end of the study (t_8_ = 9.978, *P*_8_ = 0.001) (Figure 1B).

After establishing the IAA drinking model, tail suspension test (TST), novelty-suppressed feeding (NSFT), and sucrose preference test (SPT) were used to assess depressive-like behaviors. The NSFT was used to determine whether mice in a new environment had anhedonia [24]. The latency to feed in the alcohol group was clearly higher than that in the control group (t = 12.11, *p* < 0.0001) (Figure 1C), and significantly decreased the food intake (t = 4.756, *p* = 0.0002) (Figure 1D). Traditionally, immobility was perceived as an abandonment behavior that can be expressed as depression in the TST. As shown in Figure 1E, alcohol exposure significantly increased the immobility time of mice (t = 3.051, *p* = 0.0069). Moreover, the sucrose preference was investigated as a possible indicator of depressed behavior. By contrast, the sucrose preference was lower in the alcohol group (t = 3.361, *p* = 0.0035) (Figure 1F).

**Figure 2:**
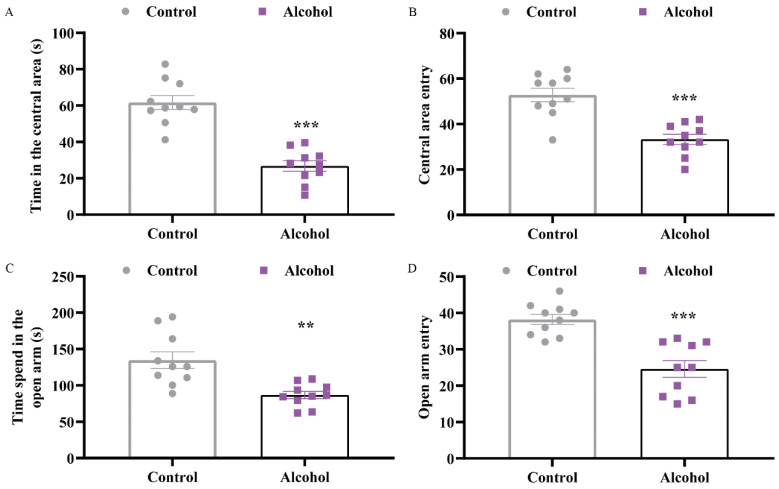
Alcohol exposure induced anxiety-like behaviors in mice. (**A**) The time of central area entries in open field test, (**B**) the time in the central area in open field test, (**C**) the number of entries in the open arms in zero maze test, and (**D**) the time spent in the open arms in zero maze test. Data are presented as the mean ± SEM, *n* = 10 for per group. Statistical analyses were carried out by Student’s *t* test, ** *p* < 0.01, *** *p* < 0.001 vs. control group.

The open field test (OFT) and zero maze test (ZMT) were performed to measure anxiety-like behaviors. Compared with the control group, the times of central area entry of mice were clearly reduced (t = 7.235, *p* < 0.0001) (Figure 2A). Mice in the alcohol group, on the other hand, spent significantly less time in the open field’s middle section (t = 5.225, *p* < 0.0001) (Figure 2B). In the ZMT, the alcohol group had remarkably less entries and spent much less time in the open arms than the control group (t_entry_ = 5.084, *p* < 0.0001; t_time_ = 3.829, *p* = 0.0012) (Figure 2C,D). These data implied that alcohol exposure led to depressive and anxiety-like behaviors.

### 2.2. Alcohol Exposure Downregulated the Expression of Synapse-Related Proteins in the Hip and PFC

**Figure 3:**
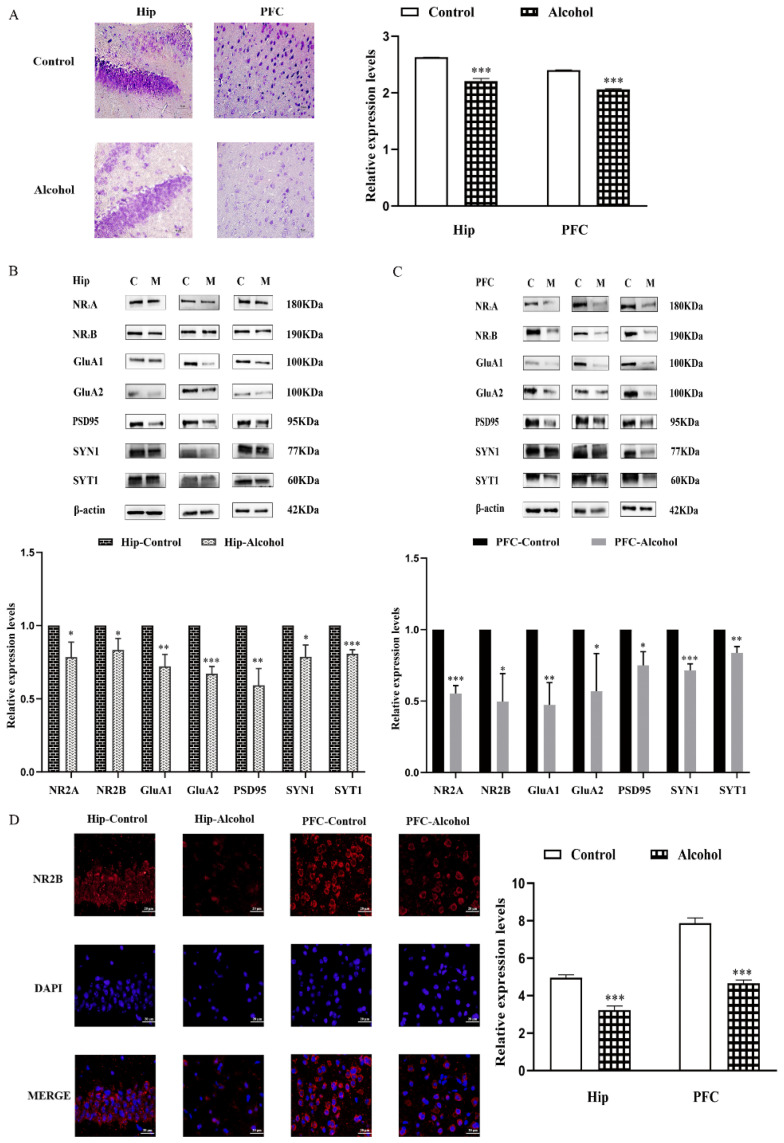
Alcohol exposure downregulated the expression of synapse-related proteins in the hippocampus (Hip) and prefrontal cortex (PFC). (**A**) Nissl staining of Hip and PFC (magnification: 400×), (**B**) Western blot analyses for synapse-related proteins in Hip, (**C**) western blot analyses for synapse-related proteins in PFC, and (**D**) immunohistochemistry (IHC) showing *N*-methyl-d-aspartic acid receptor 2B (NR2B) (red) staining in the Hip and PFC (magnification: 400×), nuclei are stained with diamidino-2-phenylindole (DAPI) (blue). Data are presented as the mean ± SEM. Statistical analysis was carried out by Student’s *t* test, * *p* < 0.05, ** *p* < 0.01, *** *p* < 0.001 vs. control group.

To explore the mechanism behind depressive-like and anxiety-like behavior induced by alcohol exposure, the histology changes in Hip and PFC were analyzed by nissl staining. There were a great number of neurons in Hip and PFC of the control group, which were neatly and densely arranged. However, the numbers of neurons were decreased in the alcohol group, a process characterized by neuron shrinkage with wider intercellular spaces than that in the control group (t_Hip_ = 15.71, *p* < 0.0001; t_PFC_ = 43.92, *p* < 0.0001) (Figure 3A).

Then, the expressions of synapse-related proteins in the Hip and PFC were analyzed by western blot. In the alcohol group, the NMDAR subunit NR2A and NR2B levels were significantly lower than in the control group (t_NR2A_ = 3.664, *p* = 0.0215; t_NR2B_ = 3.657, *p* = 0.0216) (Figure 3B,C). Meanwhile, the expressions of AMPAR subunit GluA1 and GluA2 decreased remarkably (t_A1_ = 5.908, *p* = 0.0041; t_A2_ = 11.65, *p* = 0.0003). Additionally, the postsynaptic protein PSD95 expression level was greatly reduced in the alcohol group’s hip (t = 6.179, *p* = 0.0035), we also detected downregulated expressions of presynaptic molecules, including synapsin-1 (SYN1) and synaptotagmin-1 (SYT1) in the alcohol group (t_SYN1_ = 4.562, *p* = 0.0103; t_SYT1_ = 12.05, *p* = 0.0003). Furthermore, the hippocampus sections were stained for NR2B and counter-stained with diamidino-2-phenylindole (DAPI) (Figure 3D). The alcohol group had lower levels of NR2B protein expression (t_Hip_ = 11.31, *p* = 0.0003; t_PFC_ = 16.89, *p* < 0.0001). Similar results were also found in the PFC (t_NR2A_ = 13.98, *p* = 0.0002; t_NR2B_ = 4.469, *p* = 0.0111; t_A1_ = 5.880, *p* = 0.0042; t_A2_ = 2.834, *p* = 0.0472; t_PSD95_ = 4.538, *p* = 0.0105; t_SYN1_ = 10.780, *p* = 0.0004; t_SYT1_ = 6.475, *p* = 0.0029). These results indicated that the changes in synapse-related protein expression in the hip and PFC may play a role in the behaviors impacted by chronic alcohol exposure.

### 2.3. Ferroptosis Pathway Was Involved in Alcohol-Induced Mental Disorder

**Figure 4:**
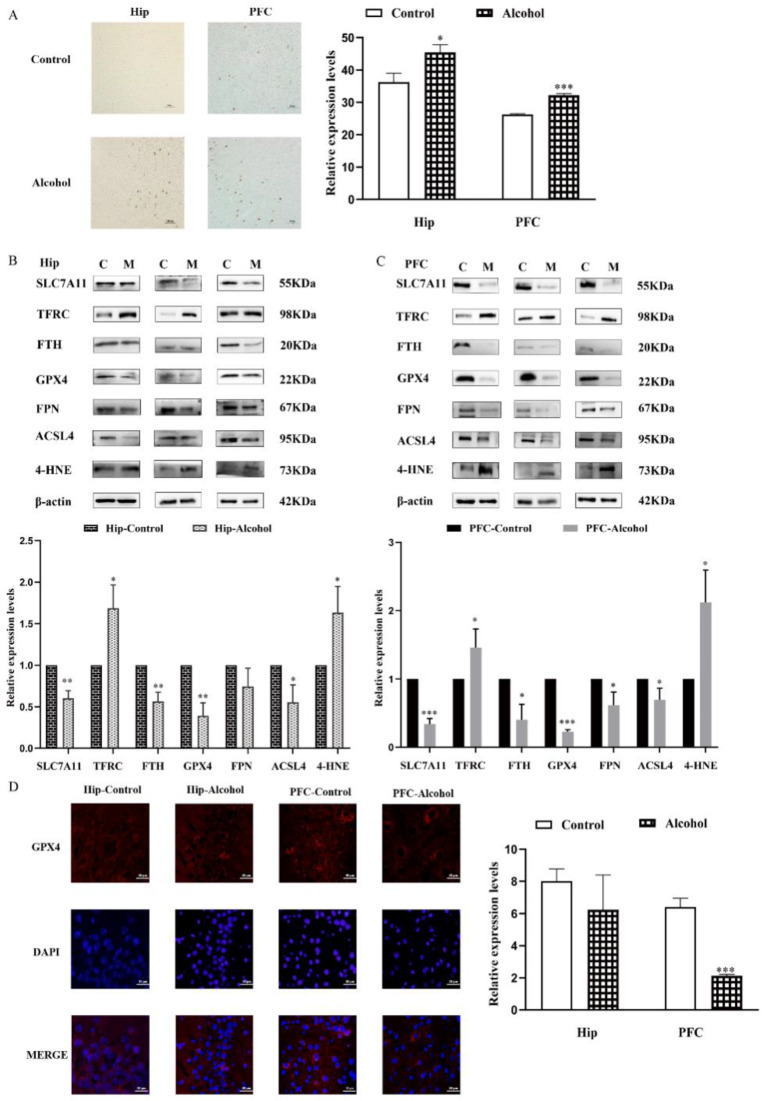
The ferroptosis pathway was involved in alcohol-induced mental disorder. (**A**) Iron staining of hippocampus (Hip) and prefrontal cortex (PFC) (magnification: 400×), (**B**) western blot analyses for ferroptosis-related proteins in Hip, (**C**) western blot analyses for ferroptosis-related proteins in PFC, and (**D**) immunohistochemistry (IHC) showing glutathione peroxidase 4 (GPX4) (red) staining in the Hip and PFC (magnification: 400×), nuclei are stained with diamidino-2-phenylindole (DAPI) (blue). Data are presented as the mean ± SEM. Statistical analysis was carried out by Student’s *t* test, * *p* < 0.05, ** *p* < 0.01, *** *p* < 0.001 vs. control group.

To prove whether the ferroptosis pathway was involved in the depressive- and anxiety-like behaviors induced by alcohol, the iron accumulation in Hip of mice was detected using iron staining. When compared with the control group, the positively stained regions in the alcohol group were slightly higher (t_Hip_ = 4.302, *p* = 0.0126; t_PFC_ = 3.728, *p* = 0.0203) (Figure 4A). In addition, the expressions of ferroptosis-related proteins in the Hip were analyzed by western blot. Significantly enhanced expressions of transferrin receptor (TFRC) (t = 4.203, *p* = 0.0137) and 4-hydroxynonenal (4-HNE) (t = 3.497, *p* = 0.0250) were characterized in the alcohol group, while alcohol markedly downregulated the expressions of glutathione peroxidase 4 (GPX4), solute carrier family 7 member 11 (SLC7A11), ferritin heavy chain (FTH), acyl-CoA synthetase family member 4 (ACSL4), and ferroportin (FPN) (t_GPX4_ = 6.738, *p* = 0.0025; t_7A11_ = 7.575, *p* = 0.0016; t_FTH_ = 6.862, *p* = 0.0024; t_ACSL4_ = 3.676, *p* = 0.0213; t_FPN_ = 2.012, *p* = 0.1146) (Figure 4B). Similar results were found in the PFC (t_TFRC_ = 2.900, *p* = 0.0441; t_4-HNE_ = 4.108, *p* = 0.0148; t_GPX4_ = 43.600, *p* < 0.0001; t_7A11_ = 14.070, *p* = 0.0001; t_FTH_ = 4.554, *p* = 0.0104; t_ACSL4_ = 3.131, *p* = 0.0352; t_FPN_ = 3.471, *p* = 0.0256) (Figure 4C). The results of immunohistochemistry also demonstrated the alcohol exposure induced a significant decline in protein expression of GPX4 in the Hip (t = 1.344, *p* = 0.2501) and PFC (t = 10.950, *p* = 0.0004) (Figure 4D). These findings suggested that ferroptosis may be involved in anxiety and depressive behaviors induced by alcohol exposure.

### 2.4. Fer-1 Improved Alcohol-Induced Cell Death In Vitro

**Figure 5:**
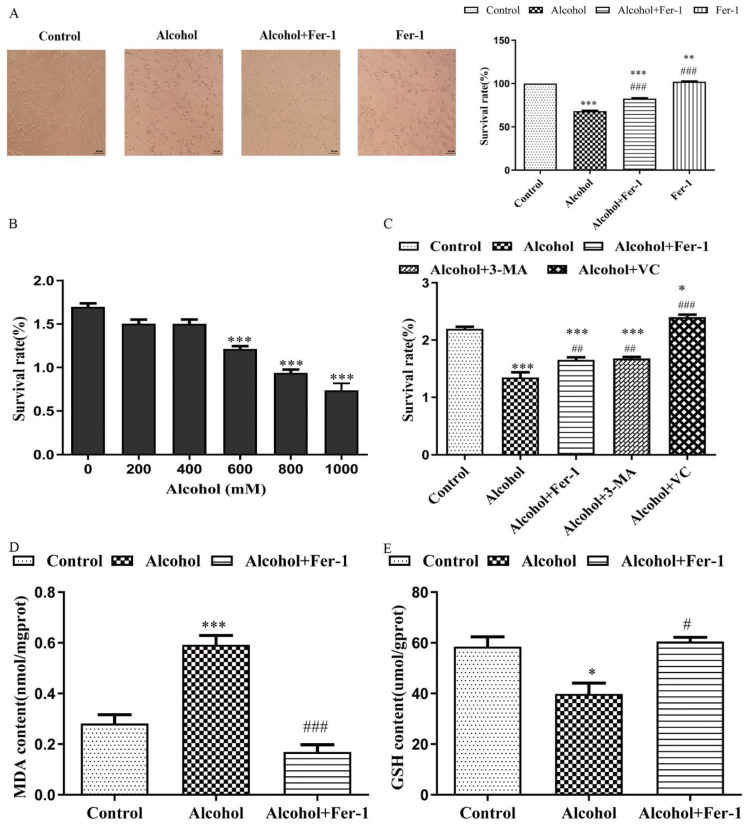
Fer-1 improved alcohol-induced cell death in vitro. (**A**) The effect of alcohol and ferrostatin-1 (Fer-1) on primary cultured neural cells, (**B**) cytotoxicity of alcohol to Neuro 2a (N2A) cells, (**C**) cytotoxicity of different inhibitors to N2A cells, Fer-1 (10 μM), 3-methyladenine (3-MA) (1 mM), vitamin C (VitC) (200 μM), (**D**) the level of lipid peroxides malondialdehyde (MDA) in N2A cells, (**E**) the level of L-glutathione (GSH) in N2A cells. Data are presented as the mean ± SEM. Statistical analysis was carried out by one-way ANOVA for multiple comparisons with Tukey post hoc test, * *p* < 0.05, ** *p* < 0.01, *** *p* < 0.001 vs. control group; # *p* < 0.05, ## *p* < 0.01, ### *p* < 0.001 vs. alcohol group.

First, the 3-(4,5-Dimethylethiazol-2-*yl*)-2,5-dlphenyl tetrazolium bromide (MTT) assay was used to verify cell viability after treatment with different concentrations of alcohol in Neuro 2a (N2A) cells (Figure 5A). Compared with the control group, the cell survival rates were 55.26% after 800 mmol/L alcohol treatment in N2A cells (t = 13.490, *p* < 0.0001). Alcohol treatment also caused the same change to primary cultured neural cells. Morphological testing showed that there were obvious synaptic connections between primary cultured neurons in the normal group (t = 49.35, *p* < 0.0001), whereas this connection was damaged in the alcohol group (Figure 5B). Then, the kinds of cell death that occurred during alcohol-induced in N2A cells were investigated using autophagy inhibitor 3-methyladenine (3-MA), ferroptosis inhibitor ferrostatin-1 (Fer-1), and antioxidant vitamin C (VitC). Interestingly, in cells treated with Fer-1 (t_primary cultured neuron_ = 5.583, *p* = 0.0014; t_N2A cells_ = 3.047, *p* = 0.0159) and VitC (t_N2A cells_ = 11.210, *p* < 0.0001), the reduced cell viability caused by alcohol (800 mmol/L) was greatly reversed, except in 3-MA (t_N2A cells_ = 3.517, *p* = 0.0079), indicating that ferroptosis may be the main form of cell death induced by alcohol (Figure 5C). Since previous studies have shown that the induction of the accumulation of lipid peroxide was the main character of ferroptosis [25,26], we detected the malondialdehyde (MDA) levels and L-glutathione (GSH) levels in N2A cells treated with alcohol and alcohol plus Fer-1. Fer-1 greatly decreased the amount of lipid peroxidation caused by alcohol, as predicted (t _alcohol, alcohol + Fer-1_ = 8.535, *p* = 0.0004) (Figure 5D). Cells could cope with excessive lipid peroxides being generated by GSH and GSH-utilizing enzymes [27]. The GSH levels in N2A cells were significantly reduced after alcohol treatment (t = 3.247, *p* = 0.0315) and vastly increased by Fer-1 (t = 4.559, *p* = 0.0103) (Figure 5E). These results demonstrated that cell ferroptosis caused by alcohol might be one of the reasons for the decreased cell survival rate.

### 2.5. Fer-1 Prevents Alcohol-Induced Ferroptosis in Cells

**Figure 6:**
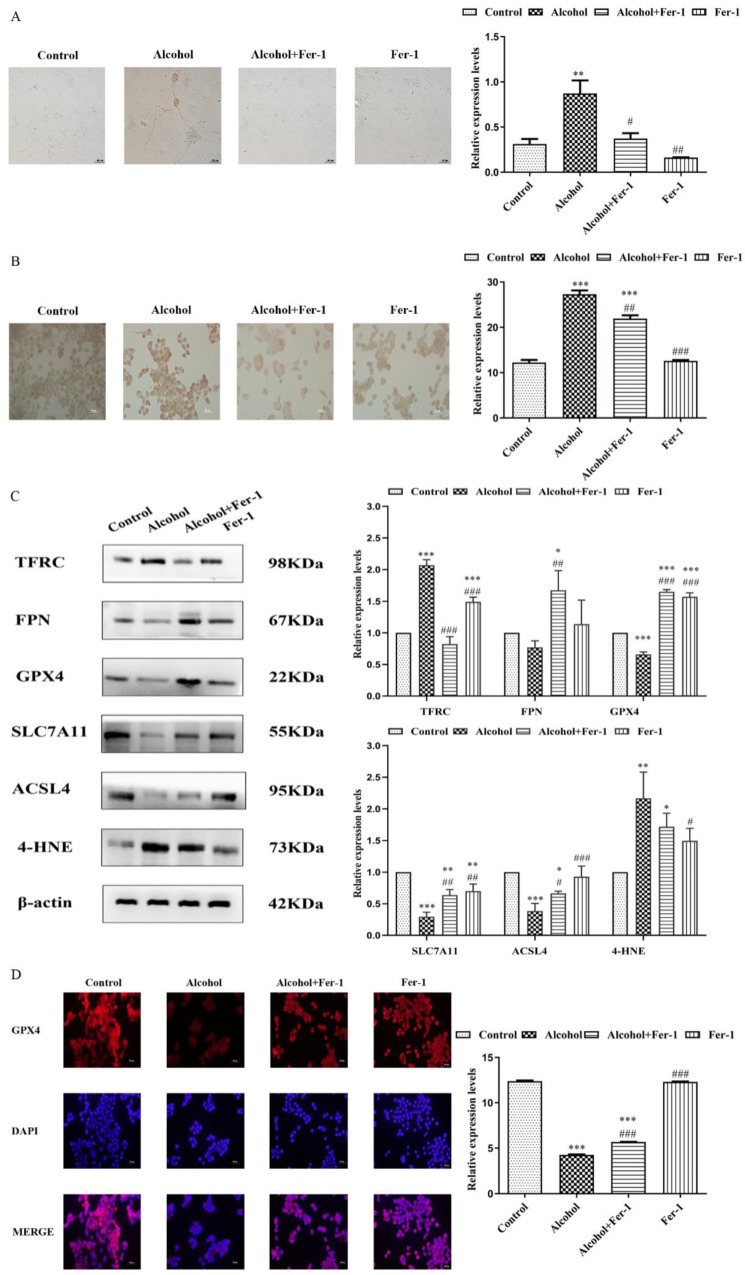
Fer-1 prevents alcohol-induced ferroptosis in cells. (**A**) Iron staining of primary cultured neuron (magnification: 400×), (**B**) iron staining of Neuro 2a (N2A) cells (magnification: 400×), (**C**) western blot analyses for ferroptosis-related proteins in N2A cells, and (**D**) immunohistochemistry (IHC) showing glutathione peroxidase 4 (GPX4) (red) staining in the N2A cells (magnification: 400×), nuclei are stained with diamidino-2-phenylindole (DAPI) (blue). Data are presented as the mean ± SEM. Statistical analysis was carried out by one-way ANOVA for multiple comparisons with Tukey post hoc test, * *p* < 0.05, ** *p* < 0.01, *** *p* < 0.001 vs. control group; # *p* < 0.05, ## *p* < 0.01, ### *p* < 0.001 vs. alcohol group.

To further verify the cell ferroptosis induced by alcohol, the primary cultured neuron and N2A cells treated with alcohol and/or Fer-1 were assessed by iron staining. We found that the positive stain areas and the integrated absorbance of iron staining in the alcohol-only treated group were both significantly increased compared with the control group of primary cultured neuron (F = 6.690, *p* = 0.0065) and N2A cells (F = 23.980, *p* < 0.0001). However, Fer-1 significantly decreased iron accumulation in contrast with the alcohol-only treated group (Fprimary cultured neuron = 5.961, *p* = 0.0125; F_N2A_ = 8.556, *p* = 0.0014) (Figure 6A,B). In addition, Fer-1 significantly increased the expressions of GPX4 (F = 42.470, *p* < 0.0001), SLC7A11 (F = 7.494, *p* = 0.0032), ACSL4 (F = 4.663, *p* = 0.0431) and FPN (F = 6.274, *p* = 0.0094) compared to the alcohol group of N2A cells. Alcohol alone significantly expanded the protein expression of TFRC (F = 23.090, *p* < 0.0001) and 4-HNE (F = 8.008, *p* = 0.0021) in N2A cells compared to the control group, while Fer-1 significantly decreased the levels of these proteins (F_TFRC_ = 26.910, *p* < 0.0001, F_4-HNE_ = 3.085, *p* = 0.207)) (Figure 6C). At the same time, the alcohol-only treated group had decreased GPX4 levels in contrast with the control group (F = 151.200, *p* < 0.0001), as determined by immunohistochemistry, and that number of cells positive for GPX4 was increased in the alcohol plus Fer-1 group compared to the alcohol-only treated group (F = 26.630, *p* < 0.0001) (Figure 6D).

### 2.6. Inhibition of Ferroptosis Ameliorated Synaptic Plasticity

**Figure 7:**
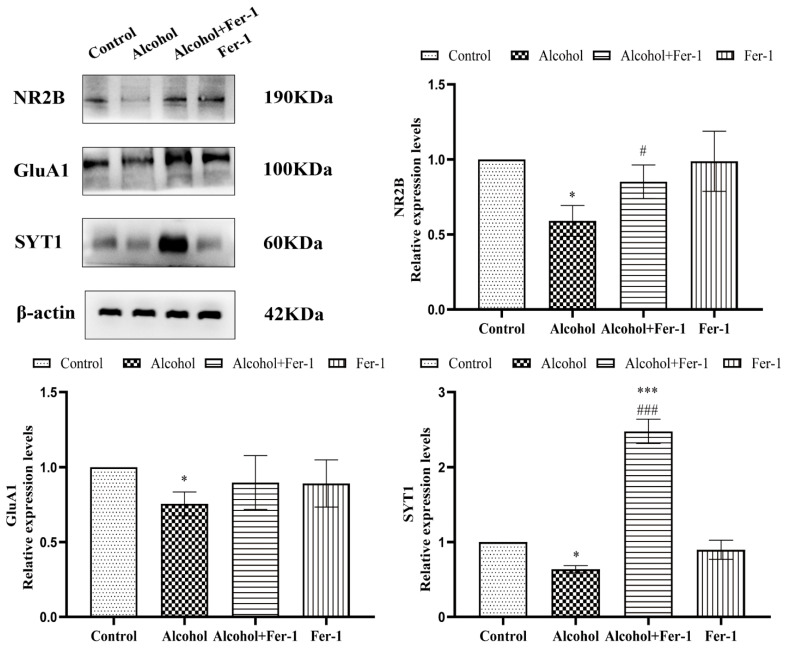
Inhibition of ferroptosis ameliorated synaptic plasticity. Protein expressions of NR2B, GluA1, and SYT1 were analyzed by Western blot. Data are presented as the mean ± SEM. Statistical analysis was carried out by one-way ANOVA for multiple comparisons with Tukey post hoc test, * *p* < 0.05, *** *p* < 0.001 vs. control group; # *p* < 0.05, ### *p* < 0.001 vs. alcohol group.

To evaluate the synaptic plasticity in N2A cells exposed to alcohol and the protective effect of Fer-1, the protein expressions of NR2B, GluA1, and SYT1 were investigated by Western blot analysis. As shown in Figure 7, the protein expressions of NR2B (F = 5.645, *p* = 0.0168), GluA1 (F = 3.370, *p* = 0.158), and SYT1 (F = 6.001 *p* = 0.0121) in N2A cells treated with alcohol were significantly decreased compared with the control group. By contrast, in comparison to the alcohol-only treated group, Fer-1 raised these protein levels (F_NR2B_ = 3.600, *p* = 0.1261; F_A1_ = 1.956, *p* = 0.5421; F_SYT1_ = 30.330, *p* < 0.0001). The above results indicated that ferroptosis was involved in the effects of alcohol exposure on synaptic plasticity.

## 3. Discussion

**Figure 8:**
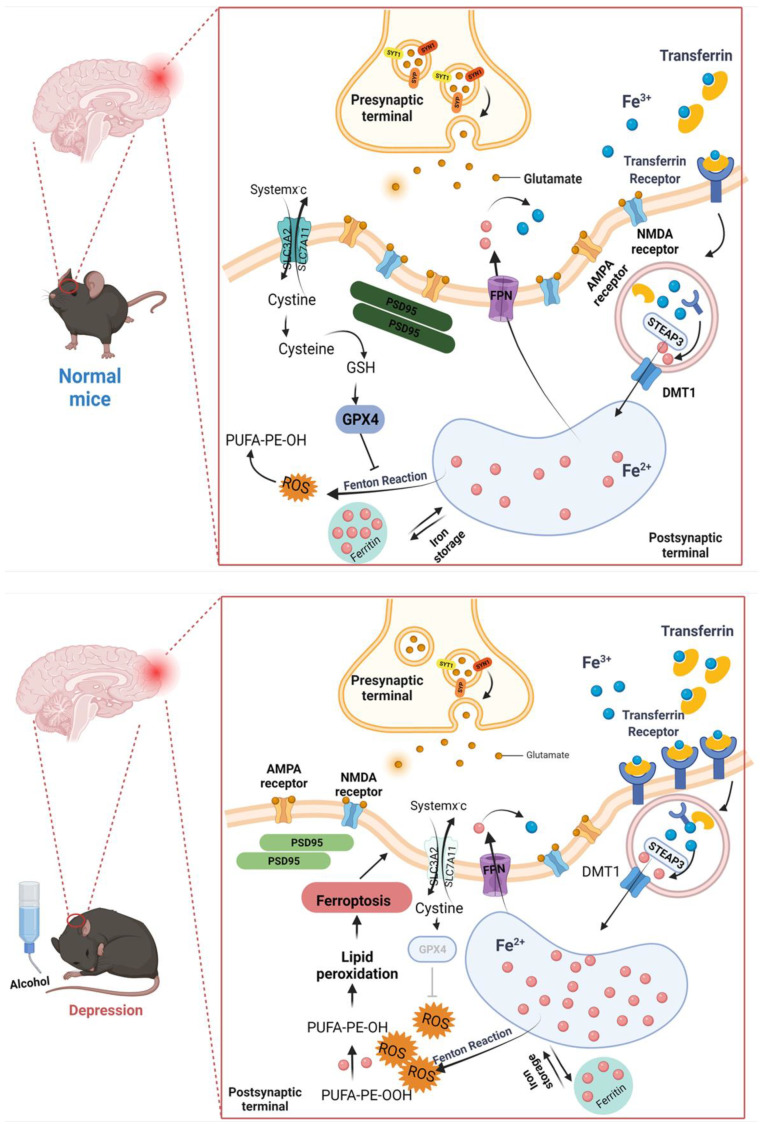
Mechanisms of ferroptosis that were involved in the depressive and anxiety-like behaviors in mice induced by alcohol exposure.

AUD is considered as a chronic recurrent encephalopathy and is the most prevalent mental disorder in the world [28]. The withdrawal symptoms of AUD showed impaired physical function and negative emotional responses, such as hypothymergasia and anxiety [29]. This comorbidity increases the complexity of diagnosis and treatment [30]. The current medical therapies, including selective serotonin reuptake inhibitors (SSRIs), tricyclic antidepressants, benzodiazepines, prazosin, etc., can hardly be regarded as effective therapeutic methods [31]. It took 3–6 weeks for drug treatment to improve AUD patients’ mood, and about 50% of patients with depression could not be cured, even suffering repeated outbreaks [32]. It reminded us that AUD comorbidity with depression and anxiety might have other more complex biological mechanisms. As a result, a deeper understanding of the processes behind alcohol-induced depressive and anxiety-like behaviors is critical for developing innovative treatment options. In this study, we found that ferroptosis may be involved in depressive and anxiety-like behaviors during early abstinence. Ferroptosis inhibitor ferrostatin-1 significantly prevented alcohol-induced neuron death in vitro. Figure 8 shows the mechanism of ferroptosis that were involved in the depressive and anxiety-like behaviors in mice induced by alcohol exposure.

Long-term alcohol consumption leads to changes in the nervous system that can lead to anxiety and depression. Hence, repeated, intermittent, and excess drinking of alcohol can generate changes in the nervous system that produce negative emotions [33]. The mice ingested 575.60 g/kg of alcohol during 8 weeks in our investigation, which was much more than the 430 g/kg reported in the literature [2]. Our results demonstrated that alcohol consumption and alcohol preference increased significantly with increasing drinking days (Figure 1A,B). Meanwhile, mice were subjected to different behavioral experiments and performed depressive-like behaviors in TST and SPT experiments and anxiety-like behaviors in ZMT and OFT testing at 3 days of forced abstinence (Figure 1C,D). The appearance of negative emotional behaviors in diverse alcohol exposure models was verified by our findings [34,35], indicating a variation in the emergence and recovery of these behaviors. Nevertheless, there was variation in NSF testing. After 7 weeks of IAA, S. Bloch et al. conducted EZM testing and NSFT testing at 24 h and 27–28 days of abstinence, respectively; the results showed that the only significant difference existed in the EZM [2]. This showed that the timing of testing during alcohol withdrawal appears to be a critical component, with negative affective behaviors appearing at certain time periods but not others, indicating that depressive-like behaviors generated by alcohol may improve with time accumulation.

The role of the Hip in the mediation of behavioral deficits triggered by alcohol was supported by the finding that human adolescents with AUD have a marked reduction in the volume of the Hip [36,37]. Ethanol administration reduced hippocampal neurogenesis [38] and inhibited hippocampal synaptic plasticity mechanisms underlying memory, resulting in long-term depression (LTD) [39,40]. The PFC is a critical neural region for mood regulation, and Kober confirmed that chronic alcohol consumption impairs the ability to cope with aversive emotional states, leading to difficulty in mood regulation [41]. Our dates showed that, compared with the control group, the number of neurons was fewer in the Hip and PFC of the alcohol group (Figure 3A). The pathogenesis of depression involves various factors such as biochemistry, psychological environment and social family, among which biological factors mainly include neuroplasticity, genes, monoamine hypothesis, endocrine, neurogenesis and so on. Jesulola E. et al. asserts that environmental factors and genetic genes cause immune and endocrine disorders; these lead to structural and functional changes in brain regions, which then result in neurogenesis disorders, synaptic formation disorders and neurotransmission dysfunction, and eventually the human body begins to show symptoms of depression [42]. At present, one of the mainstream studies into the pathogenesis of depression concerns the hypothesis of glutamatergic system dysfunction [43,44,45].

Several studies have demonstrated that alcohol alters synaptic transmission and plasticity in some brain regions involved in the addiction cycle [46,47,48]. Specifically, chronic intermittent exposure to alcohol disrupts the NR2B proteome and regulates the (mGlu)_1/5_ receptor-dependent long-term depression in mice [17]. Many studies have focused on other brain regions, such as the striatum and nucleus accumbens [49,50]. Therefore, we investigated the effects of IAA-induced depressive and anxiety-like behaviors on synaptic transmission and plasticity in the hip and PFC of adult mice. We observed significantly reduced expression levels of NMDAR subunit proteins (NR2A/2B) and AMPAR subunit proteins (GluA1/A2) in the alcohol group. Furthermore, we found the levels of synapse-related proteins (SYN1, SYT1) and postsynaptic protein PSD95 were significantly decreased in the hip and PFC of IAA model mice (Figure 3B–D). Taken together, these results suggested that the changes in synaptic plasticity in the hip and PFC may be involved in the behavioral effects induced by acute alcohol withdrawal.

Iron is recognized as the most abundant transition metal in the brain [51], its accumulation in the brain is spatially and temporally heterogeneous. Higher concentrations of iron within the brain are preferentially found in the nucleus accumbens, substantia nigra (SN), deep cerebellar nuclei and parts of the hippocampus [52,53]. Acting as a protein cofactor, iron delivery to the brain is essential for multiple neurological processes including myelination, neurotransmitter synthesis, synaptogenesis and energy production [54,55,56]. Brain iron status is consistently maintained and tightly regulated at the level of the blood–brain barrier (BBB) [57]. In the brain, iron supply to neurons, astrocytes, and microglia depends not only on transferrin-bound iron (TFR1), but also on non-transferrin-bound iron (NTBI) via divalent metal transporter 1 (DMT1) [58,59,60].

The brain is particularly sensitive to the disruption of iron homeostasis. Iron deficiency has a negative effect on synaptic plasticity by decreasing the fraction of vesicular release in response to repetitive stimulation, causing learning and memory deficits [51]. On the other hand, high levels of iron cause a build-up of toxic reactive oxygen species that interfere with mitochondrial function [61], damage DNA [62], catalyze dopamine oxidation reactions to produce toxic quinones [63] and irreversibly modify proteins through highly reactive aldehydes [64]. All these causes of cell stress ultimately lead to iron-mediated cell death. Ferroptosis is a newly discovered type of regulated cell death that is morphologically, biochemically, and genetically distinct from apoptosis, necroptosis, and autophagy. Ferroptosis is characterized by a disruption of iron metabolism and an iron-dependent growth of lipid peroxidation, caused by inhibition of System Xc- or GPX4 [65]. Ferritin (composed of FTH and FTL, excess intracellular iron is stored in ferritin), transferrin, hepcidin, and iron-related proteins work together to maintain iron homeostasis [66].

Ferroptosis induced by alcohol consumption was found in the peripheral and other organs. In these organs, alcohol intake reduced the protein and mRNA expression levels of GPX4, SLC7A11, FTL, and FTH1 [67,68]. In recent years, ferroptosis has been proved to be closely related to the occurrence and development of various neurological disorders, such as Alzheimer’s disease (AD) [69,70,71,72], Parkinson’s disease (PD) [73,74] and Huntington’s disease (HD) [75,76,77] et al. The hallmark features of most neurodegenerative diseases are the accumulation of total iron and an unstable iron pool (LIP) in specific brain regions, and these symptoms can be ameliorated by ferroptosis inhibitors [74,78,79]. Interestingly, evidence of ferroptosis was found in the hippocampus of CUMS depression mouse model [80]. Wang et al. revealed that NaHS reduced ferroptosis in the PFC of the T1DM mouse model by reducing iron deposition and oxidant stress, thus alleviating the anxiety-like and depression-like behavior of the mouse model with T1DM [81]. These studies suggest that GPX4-mediated ferroptosis may be a potential mechanism affecting anxiety and depression [82]. However, there are few reports on the relationship between iron overload and synaptic plasticity in emotional behaviors. Therefore, it is necessary to explore whether ferroptosis is involved in the changes in synaptic plasticity caused by alcohol-induced anxiety and depression-like behaviors. Here, our histology and immunohistochemistry analysis observed iron accumulation in hip and PFC of alcohol-exposed mice (Figure 4A). Then, we found the proteins expression of GPX4, SLC7A11, FTH, ACSL4 and FPN, which were also significantly reduced in depressive and anxiety-like behavioral mice whose symptoms were induced by alcohol (Figure 4B–D). This evidence proved that ferroptosis was involved in alcohol-induced neurotoxicity. Consistent with these earlier studies, our in vitro cell experiments confirmed that alcohol treatment could lead to iron overload (Figure 6A,B), while Fer-1 treatment reversed the levels of ferroptosis-related proteins (Figure 6C).

Although the amount of iron in the brain is less than 2% of the body, it is closely related to brain development, synthesis, and neurotransmitter metabolism. The control of iron entry is tightly linked to synaptic activity [83]. Moreover, in the excitatory synapses of hippocampal neurons, iron uptake depends on the direct stimulation of NMDA receptors and indirect activation of VOCCs (by synaptic activation, back propagating action potentials or depolarization from adjacent synapses) [84,85]. In addition, as one of the iron supplies of neurons and astrocytes, NTBI is important to restore the pool of iron–sulfur clusters and thus to meet the elevate energy consumption caused by neuronal activity, but also to sustain specific neuronal functions, such as neurotransmitter synthesis. Moreover, NTBI can contribute to the “oxidative tone” which is important for both basal synaptic transmission and long-term potentiation [86]. Excessive iron can cause activated microglia to release many neuroinflammatory factors, aggravate the deformation of dopaminergic neurons, and lead to the decreased expression of synapse-related proteins AMPAR1 and PSD95 [87]. Ferroptosis is involved in the functional regulation of dopamine neurons by affecting synaptic plasticity [88]. Interestingly, our study showed that the expression of NR2B, GluA1 and SYT1 were decreased in the alcohol-only treated cells, while Fer-1 treatment reversed the levels of synapse-related proteins (Figure 7). The above results indicated that ferroptosis was involved in the effects of alcohol exposure on synaptic plasticity.

In summary, these results suggested that iron homeostasis regulated the expression of synaptic proteins in alcohol-exposed mice, thereby affecting hippocampal and prefrontal cortical synaptic plasticity, leading to difficulty in mood regulation, exhibiting expressive and anxiety-like behaviors. Therefore, inhibiting ferroptosis might be a promising strategy for the treatment of AUD.

## 4. Materials and Methods

### 4.1. Reagents and Materials

Fer-1, 3-MA were purchased from Med Chem Express Ltd. (Newark, NJ, USA). VC and DMSO were from Sinopharm chemical reagent Co, Ltd. (Shanghai, China). Anti- GluNR2B, anti-GluNR2A, anti-GluA1, anti-GluA2, anti-SYN1, anti-SYT1 and anti-PSD95 were obtained from Cell Signaling Technology (Boston, MA, USA). Anti-FPN-1, Anti-GPX4, and Anti-TFRC, anti-ACSL4, anti-4-HNE were from Abcam (Boston, MA, USA). Anti-SLC7A11, anti-β-actin, goat anti-rabbit-HRP second antibody, and MTT were purchased from Beyotime Technology (Shanghai, China). Anti-FTH1 was from Absin Technology (Shanghai, China). MDA Assay Kits and L-glutathione GSH Assay Kits were obtained from Nanjing Jiancheng Bioengineering Institute (Nanjing, China). Goat anti-mouse IgG was from Jackson ImmunoResearch (West Grove, PA, USA). Penicillin, fetal bovine serum (FBS), streptomycin, Dulbecco’s Modified Easle’s medium/F12 (DMEM/F12), and trypsin were purchased from Thermo Scientific (Waltham, MA, USA).

### 4.2. Animal Treatment

**Figure 9:**
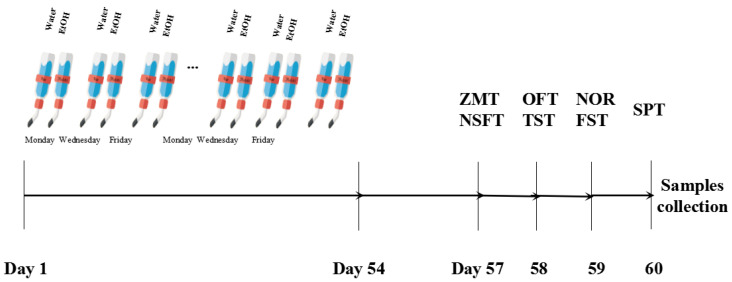
Schematic of the experimental timeline for alcohol drinking and behavioral testing.

C57BL/6 mice (Beijing Vital River laboratory; 8–9 weeks, half male and half female) were acclimated for at least 7 days before treatment. All mice were singly housed in a 12 h reverse light-dark cycle, with free access to food and water during the study. Notably, mice were divided randomly into the control group and alcohol group, and the intermittent access to alcohol (IAA) two-bottle choice drinking procedure, and behavioral testing were performed in Figure 9. Briefly, on Monday, Wednesday, and Friday, alcohol group mice were provided two bottles (single bottles of 20% ethanol and water) for 24 h across 8 weeks. Both bottles containing tap water were given to the control group. To avoid side preference, the bottle position was swapped every day. The bottles were weighed every day and mice were weighed weekly. All animal experiment procedures were approved by the Ethics Committee of Jianghan University (JHDXLL2020-009). Additionally, all methods were carried out in accordance with Jianghan University guidelines and regulations. This study is in accordance with ARRIVE guidelines for the reporting of animal experiments.

### 4.3. Behavioral Testing

During withdrawal, mice were observed for depressive and anxiety-like behavior (see Figure 1A for the experimental timeline). Before behavioral testing, mice were kept in the testing room to get habituated to the surroundings.

#### 4.3.1. Zero Maze Test

The maze is consisting of a black acrylic ring track with 10 cm diameter and 105 cm height. The maze is divided into four quadrants; the two opposite quadrants are opened zones, and the other two opposite quadrants with 20 cm high walls are closed zones. Mice were placed at a random junction of the area facing a closed zone and were permitted to freely explore for 5 min, and then were immediately removed from the maze, as mentioned earlier [89]. At the end of each test, the ZMT was cleaned with 70% alcohol. The number of entries into each zone, as well as the time and distance spent in each zone, were calculated.

#### 4.3.2. Novelty-Suppressed Feeding Test

The NSFT was conducted as previously described [90]. Mice were fasted for 24 h and then placed at one corner of an opaque square arena (40 × 40 cm), with chow in the center, on the day of testing. The mice were permitted to explore the arena for 10 min, and the first time a bite of chow was taken was recorded as the latency to feed. Meanwhile, after 10 min, the amount of chow ingested was calculated. Chow intake per unit time = amount of chow consumed in 10 min/mouse’ weight. Between each session, the arena was cleansed with 70% alcohol.

#### 4.3.3. Open Field Test

The OFT was performed as previously described [91]. Briefly, the mice were placed in the center of a 40 × 40 cm open field arena, and their behavior was monitored for 15 min. We evaluated central area entry, and time spent in the center. Between each session, the arena was cleansed with 70% alcohol.

#### 4.3.4. Tail Suspension Test

The TST was used to assess the depressive-like behavior of mice and carried out according to previous publications with minor modifications [92]. Briefly, mice were hung to 50 cm above the tabletop with a rope fixed to the tip of the tail in a sound-isolated room. The test was videotaped, and the immobility time of the mice was measured for 6 min by SuperTst software (version XR-XX203, Shanghai XinRuan information Technology Co., Ltd., Shanghai, China).

#### 4.3.5. Sucrose Preference Test

With minor modifications [93], the SPT was performed over a 24 h period using two bottles, one with 1% sucrose solution and the other with water. The mice were denied food and water for one day before the test. After 12 h, the position of the bottles was swapped to avoid any potential drinking preference. Two bottles were weighted after 24 h. The percentage of sucrose solution taken in relation to the total amount of liquid taken was used to compute the sucrose solution preference.

### 4.4. Histology

After fixation and embedding, the Hip and PFC sections, 5 μm thick, were prepared using a rotary microtome. The brain sections were rehydrated in gradient alcohol after being deparaffinized in xylene.

#### 4.4.1. Nissl Staining

After rehydrated, sections were stained with toluidine blue [94]. The samples were analyzed using an optical microscope with a 400× magnification. The cells were counted by Image J software (version 1.42q, National Institutes of Health, Bethesda, MD, USA), and the mean counts were determined.

#### 4.4.2. Iron Staining

Iron staining was performed as described previously [95]. Sections were rehydrated and then incubated in a freshly prepared solution of equal parts 4% hydrochloric acid and 4% potassium ferrocyanide for 30 min at room temperature. After washing with PBS three times, the sections were immersed in methanol containing 1% hydrogen peroxide for 20 min, washed three times with PBS, and then reacted with 3,3-diaminobenzidine (DAB) until sections appeared to have some brown granules. The reaction was terminated with distilled water, and the sections were sealed using neutral gum.

#### 4.4.3. Immunohistochemistry

The immunohistochemistry was adapted from the previous literature described [96]. After being rehydrated, sections were prepared for antigen retrieval for 20 min with filling sodium citrate buffer. The temperature of the slides was reduced to 25 °C, before being washed three times with TBS for 5 min each time. After blocking with a buffer for 25 min, the sections were incubated with primary antibodies (anti-NR2B, anti-GPX4 diluted at 1:200) overnight. Sections were treated with secondary antibodies for 1 h at 37 °C after three washes with TBS, and then sealed using the antifade mounting medium with DAPI.

### 4.5. Western Blot Analysis

The proteins of Hip and PFC were extracted from the lysis buffer containing protease and phosphatase inhibitors. The protein concentrations were estimated using a BCA Protein Assay Kit (Boster Biological Technology Co., Ltd., Wuhan, China). A total of 10 μg proteins were separated by SDS/PAGE and electroblotted onto a nitrocellulose membrane. First, the membranes were blocked with a blocking buffer for 1h at RT. Then, different primary antibodies were incubated for 12 h at 4 °C on the membranes, followed by the secondary antibody. The following antibodies were used: anti-NR2A, anti-NR2B, anti-GluA1, anti-GluA2, anti-PSD95, anti-TFRC, anti-SLC7A11, anti-FTH, anti-FPN, anti-ACSL4, anti-4-HNE, anti-β-actin (1:1000), anti-SYN1, anti-SYT1 (1:10,000), and anti-GPX4 (1:4000). Bands were visualized with the GENE GNOME SYNGENE BIO IMAGING system (version Gene Sys, Gene company, Hong Kong, China).

### 4.6. Cell Treatment

#### 4.6.1. Primary Cultures of Prefrontal Cortical Neural Cells

Prefrontal cortical neurons were isolated, as reported previously, with some modifications [97,98]. Newborn SD mice (day 0) were rapidly sacrificed by decapitation. Using an anatomical microscope, the hippocampus was separated and transferred into an ice-cold PBS. The hippocampus was then made into a single-cell suspension. Cells were resuspended by a DMEM medium, supplemented with 20% FBS, grown at a density of 1.0 × 10^7^/mL in culture dishes, and cultured in the CO_2_ incubator. The DMEM medium was discarded after 4 h and replaced with a neurobasal medium supplemented with B27. Subsequently, cells were cultured for 7 days to obtain pure and mature neurons for iron staining analysis and MTT.

#### 4.6.2. Cultures of N2A Cells

N2A cells, a mouse brain neuroma line, were purchased from the Kunming Institute of Cell Library (Kunming, China) and were cultured with DMEM/F12 medium (containing 10% FBS, 100 U/mL antibiotics penicillin, and 100 μg/mL streptomycin) in an incubator at 37 °C and 5% CO_2_. The cells were then harvested and plated in 24-well plates at a density of 3 × 10^5^ cells/well, or 6-well plates at a density of 5 × 10^4^ cells/well for iron staining analysis, immunohistochemistry analysis, and Western blot analysis as previously described.

### 4.7. Measurement of Cell Viability, MDA Contents and GSH Contents

The MTT test was used to determine cell viability [99]. Briefly, the cells were seeded on a 96-well plate at a density of 5 × 10^3^ cells/well overnight and treated with different concentrations of alcohol (200, 400, 600, 800 and 1000 mmol/L). After 24 h incubation, 20 μL of MTT (0.5 mg/mL) reagent was added to each well. The supernatants were carefully removed 4 h later, and 150 μL of DMSO was added to each well and mixed thoroughly. Then, the absorbance was measured at 570 (A_570_) nm using a microplate reader. The MDA levels and GSH levels in the cell lysates were measured using a commercial assay kit, as directed by the manufacturer.

### 4.8. Statistical Analysis

All analyses were carried out by using the GraphPad Prism (version 8.0, GraphPad Software Inc, San Diego, CA, USA), and results were reported as means ± S.E.M (standard error of the mean). Alcohol consumption and preference data were analyzed using repeated one-way measures ANOVA with Sidak’s post hoc test for multiple comparisons, an independent sample *t* test was applied to other animal experiments, and the cell experiments data were analyzed using one-way ANOVA for multiple comparisons with a Tukey post hoc test to determine statistical significance. Values of *p* < 0.05 were considered statistically significant.

## 5. Conclusions

In our conclusions, alcohol exposure and acute withdrawal could induce depressive and anxiety-like behaviors in mice, and this effect may occur via activating ferroptosis. The novelty of this study was the finding that ferroptosis takes part in the regulation of synaptic plasticity post alcohol exposure, consequently we provide a reference for the mechanism research of AUD with depression and anxiety. However, this mechanism study was limited to the cellular level and has not been verified at the animal level. More direct evidence is needed for the role of iron homeostasis in alcohol addiction and its induced anxiety and depression.

## Data Availability

Requests to access the datasets should be directed to the corresponding author.
